# Dynamics of Supramolecular Ionic Gels by Means of Nuclear Magnetic Resonance Relaxometry—The Case of [BMIM][Cl]/Propylene Carbonate Gel

**DOI:** 10.3390/molecules30122598

**Published:** 2025-06-15

**Authors:** Michał Bielejewski, Robert Kruk, Danuta Kruk

**Affiliations:** 1 Institute of Molecular Physics, Polish Academy of Sciences, M. Smoluchowskiego 17, 60-179 Poznań, Poland; michal.bielejewski@ifmpan.poznan.pl; 2 Department of Chemistry, University of Warmia and Mazury in Olsztyn, Plac Łódzki 4, 10-957 Olsztyn, Poland; robert.kruk@uwm.edu.pl; 3 Department of Physics and Biophysics, University of Warmia and Mazury in Olsztyn, Michala Oczapowskiego 4, 10-719 Olsztyn, Poland

**Keywords:** ionic gels, ionic liquids, molecular dynamics, nuclear magnetic resonance, relaxation

## Abstract

Aiming to obtain insight into the dynamic properties of ionogels, ^1^H NMR relaxation experiments were performed for an ionogel composed of 1-butyl-3-methyl-imidazolium chloride [BMIM][Cl] and propylene carbonate. The experiments were conducted in the frequency range of 10 kHz to 20 MHz, spanning the temperature range of 273 K to 338 K. The data were analyzed in term s of a relaxation model including two relaxation contributions—one of them associated with anisotropic (two-dimensional) translation diffusion, the second one representing a power law dependence of spin-lattice relaxation rates on the resonance frequency. The power law relaxation term (characterized by a very low power law factor of about 0.1) was attributed to the collective dynamics of the partially immobilized propylene carbonate matrix, while the relaxation contribution associated with anisotropic translation diffusion was attributed to the movement of BMIM cations in the matrix; the translation diffusion coefficient was estimated as varying in the range of 10^−13^ m^2^/s–10^−12^ m^2^/s. Moreover, other parameters were determined as a result of the analysis, such as the residence lifetime on the matrix surfaces. Subsequently, the temperature dependencies of the determined parameters were assessed.

## 1. Introduction

Materials engineering has emerged as a powerful tool for designing and producing novel materials with enhanced properties tailored to specific applications. For instance, by combining systems with distinct characteristics, one can create new materials that display the strengths of each component while minimizing their individual drawbacks. The solidification process is one example of such an approach, improving the mechanical, processibility and safety properties of technologically important liquids. In recent years, sustainability has also become a very demanded property, prompting material engineers to design systems that can be easily recoverable and have low or no impact on the natural habitat. To fulfil these requirements, low molecular weight gelators have been used to solidify different liquids and solutions. These gelators are derivatives of monosaccharides, cholesterols, hydrazides, and other materials, being neutral to the environment and having good biodegradability [1,2,3,4,5]. The most affected property during the solidification process is the significant reduction in the dynamics of liquids confined within the polymeric networks. This can limit the kinetics of technologically important processes, e.g., purification, synthesis, analysis, catalysis. The gelators can solidify a vast range of different liquids in a hierarchically organized self-assembly process macroscopically expressed in gel formation. This process is driven by non-covalent interactions such as the electrostatic interaction, van der Waals interaction, π–π stacking, coordination, London forces, and hydrogen bonds. Designing the chemical structure of low molecular weight gelators and equipping them with functional moieties such as aromatic rings, hydrophobic and hydrophilic parts, electron donors, and acceptor groups allows for the simultaneous activation of different non-covalent interactions that influence the self-assembly phenomena during the gelation process. Such an approach proved to be a valuable tool in developing well-defined nanostructures. In the literature, a vast number of papers have shown the possibility of using physical gels in a variety of different fields of industry (in pharmaceutics, medicine, engineering, electronics, etc.) [6,7,8,9,10,11,12,13,14,15]. Especially interesting seems to be the possibility of adjusting the chemical structure of gelators so they can solidify specific types of liquids such as polar, non-polar, organic, water, oils, and emulsions. Among them also are ionic liquids, defined as compounds entirely composed of ions, with melting points below 100 °C, which are considered as engineering fluids that can be used in green chemistry. Based on their variety of chemical structures, ionic liquids can be divided into different categories, e.g., room-temperature ionic liquids [16,17,18,19,20,21], task-specific ionic liquids [22,23], polyionic liquids [24,25]. The wide structural diversity of ionic liquids, along with their varied intermolecular interactions, gives rise to a complex range of interfacial phenomena within the gel system. Extensive studies of these materials have improved the understanding of the self-organization of gelator molecules into a three-dimensional network structure, the gelation phenomenon, and the dependence of the thermal stability and gel morphology on the gelation solvent [26,27,28,29,30,31,32,33,34,35].

In parallel to the development of advanced materials, there is a need for experimental and theoretical methods giving insight into the structural and dynamic properties of such materials with the purpose of establishing links between their molecular properties and functional features. Nuclear magnetic resonance (NMR) relaxometry plays a vital role in such investigations. Typically, NMR relaxation experiments are performed over a single (high) magnetic field (resonance frequency). Thanks to fast field cycling (FFC) technology, it is possible to conduct relaxation experiments over a very broad range of resonance frequencies, at least from 10 kHz to 10 MHz (often higher). The possibility of varying the magnetic field has introduced a new dimension to NMR relaxometry, leading to unique advantages for this method. The intrinsic property of relaxation processes, originating from the quantum–mechanical foundation of spin relaxation, is that at a given resonance frequency the most efficient relaxation mechanism is associated with dynamic processes occurring on a timescale matching the reciprocal resonance frequency. This implies that by varying the resonance frequency, one can probe in a single experiment dynamic processes occurring on much different timescales, beginning with slow dynamics over low magnetic fields and then moving to progressively faster motion. Moreover, the shape of the frequency dependencies of the spin-lattice relaxation rates (reciprocal relaxation times) reflects the mechanism of the molecular and ionic motion; for instance, one can distinguish between isotropic and anisotropic rotational and translational motions, sub-diffusive dynamics, or polymer-specific motion [36,37,38,39,40,41,42,43]. The relaxation rates are linked to the dynamic properties via quantities referred to as spectral density functions (Fourier transforms of corresponding time correlation functions). The mathematical forms of the spectral density functions are motion-specific [36,37,38,44]. Consequently, the character of the motion is imprinted in the shape of the frequency dependence of the relaxation rates. This means that FFC NMR relaxation experiments have the potential to reveal not only the timescale of the dynamic processes but also their mechanisms. This exceptional ability has been exploited in investigating dynamic properties of molecular and ionic systems of various complexities, from liquids, via macromolecules, tissues, food products, confined and porous systems, to solids [45,46,47,48,49,50,51,52,53].

In this work, we exploit FFC NMR relaxometry to investigate the dynamics of ionogels, using a system including 1-butyl-3-methyl-imidazolium chloride ionic liquid and propylene carbonate as an example.

## 2. Results

### 2.1. NMR Relaxation Theory

The predominant source of ^1^H spin-lattice relaxation is ^1^H-^1^H magnetic dipole–dipole interactions. The interactions fluctuate in time as a result of molecular (ionic) motion. According to the spin relaxation theory [54,55], the spin-lattice relaxation rate, $R_{1}\left(\omega \right)$ (ω denotes the ^1^H resonance frequency in angular frequency units), is given as a sum of the spectral density functions:

(1) $$R_{1}\left(\omega \right)=C\left[J\left(\omega \right)+4J\left(2\omega \right)\right]$$

where *C* denotes a dipolar relaxation constant. The spectral density function, $J\left(\omega \right)$, is a Fourier transform of the corresponding time correlation function characterizing the motion causing the fluctuations of the dipole–dipole coupling. The mathematical form of the correlation function, and consequently the spectral density, depends on the mechanism of the motion. Moreover, one can expect several relaxation contributions associated with different mechanisms of the fluctuations of dipole–dipole interactions. Anticipating the results, the spectral density function describing two-dimensional (2D) diffusion (also referred to as surface diffusion) has the following form [36,37,38,39,40]:

(2) $$J_{2D}\left(\omega \right)=\tau_{trans}ln\left[\frac{1+{\left(\omega \tau_{trans}\right)}^{2}}{{\left(\frac{\tau_{trans}}{\tau_{res}}\right)}^{2}+{\left(\omega \tau_{trans}\right)}^{2}}\right]$$

where the correlation time, $\tau_{trans}$, is defined as $\tau_{trans}=\frac{d^{2}}{2D_{trans}}$ [56,57,58], $D_{trans}$ denotes the translation diffusion coefficient of the diffusing molecule (ion), d is its diameter (under the approximation of a spherical shape), and $\tau_{res}$ is a residence lifetime of water of the macromolecule on the surface. For $\omega \tau_{trans}\ll 1$ and $\omega \tau_{res}\gg 1$ (the conditions imply that $\tau_{res}\gg \tau_{trans}$), the spectral density (and consequently the corresponding relaxation rate) shows a linear dependence on the logarithm of the resonance frequency, ω. Using Equations (1) and (2), the overall relaxation rates for the ionogel can be expressed as:

(3) $$\begin{array}{c} R_{1}\left(\omega \right)=R_{1}^{2D}\left(\omega \right)+R_{1}^{power-law}\left(\omega \right)=\\ {\;C}_{trans}\tau_{trans}\left[ln\left[\frac{1+{\left(\omega \tau_{trans}\right)}^{2}}{{\left(\frac{\tau_{trans}}{\tau_{res}}\right)}^{2}+{\left(\omega \tau_{trans}\right)}^{2}}\right]+4ln\left[\frac{1+{\left(\omega \tau_{trans}\right)}^{2}}{{\left(\frac{\tau_{trans}}{\tau_{res}}\right)}^{2}+{\left(2\omega \tau_{trans}\right)}^{2}}\right]\right]+A\omega^{-\alpha } \end{array}$$

The relaxation contribution $R_{1}^{2D}\left(\omega \right)$ orresponds to the 2D translation diffusion ($C_{trans}$ denotes the dipolar relaxation constant associated with the diffusion process), while the relaxation term $R_{1}^{power-law}\left(\omega \right)$ describes a relaxation contribution described as a power law dependence of the relaxation rates on the resonance frequency, with a phenomenological pre-factor, A, and a power law factor, α.The unit of A depends on the value of α. The power law dependencies of spin-lattice relaxation rates are characteristic of polymer and protein motion [47,48,49,50,51,52,59,60,61,62,63,64,65,66,67,68].

### 2.2. Analysis

**Figure 1:**
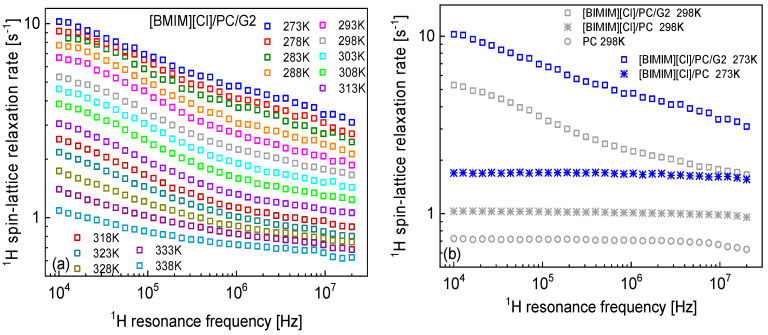
(**a**) ^1^H spin-lattice relaxation data for [BMIM][Cl]/PC/G2 versus temperature. (**b**) Comparison of ^1^H spin-lattice relaxation data for [BMIM][Cl]/PC/G2, [BMIM][Cl]/PC (solution) and bulk PC at 273 K and [BMIM][Cl]/PC/G2 and [BMIM][Cl]/PC (solution) at 298 K.

The ^1^H spin-lattice data collected for the ionogel in the temperature range from 273 K to 338 K are shown in Figure 1a. The data are complemented with ^1^H spin-lattice relaxation rates for [BMIM][Cl]/PC (without G2) and bulk PC at 298 K and [BMIM][Cl]/PC (without G2) at 298 K. The gel preparation is described in Section 4; here, we explain the abbreviations for 1-butyl-3-methyl-imidazolium chloride ionic liquid ([BMIM][Cl]), propylene carbonate (PC), and cyclo(L-beta-2-ethylhexylasparaginyl-L-phenylalanyl) (G2).

It is worth noting that the relaxation data for [BMIM][Cl]/PC (without G2) and bulk PC are (based on a good approximation) frequency-independent, indicating fast dynamics. Independently of the form of the spectral density function that one might assume, the frequency independence means that the condition $\omega \tau_{c}\ll 1$ (where $\tau_{c}$ denotes a correlation time) applies.

**Figure 2:**
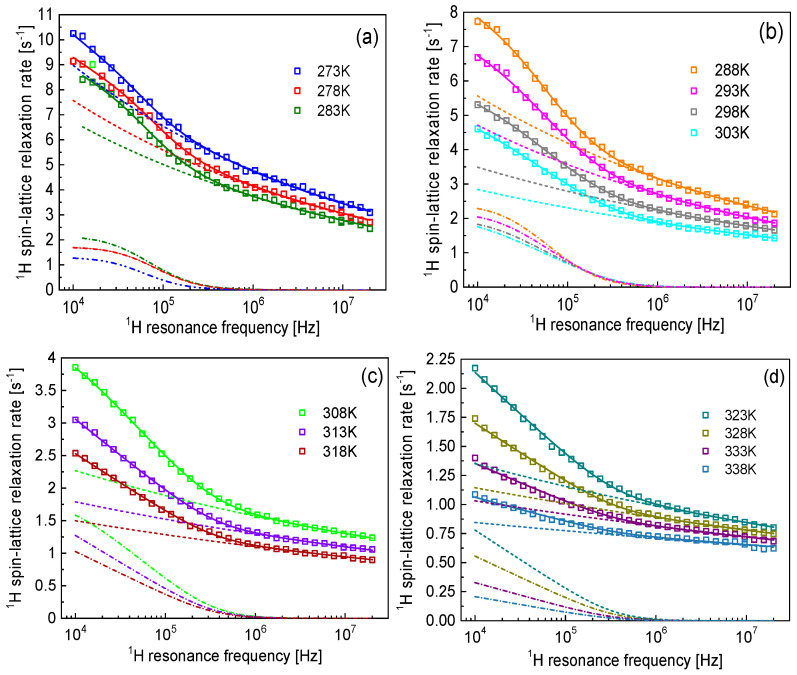
^1^H spin-lattice relaxation rates for [BMIM][Cl]/PC/G2 versus temperature ((**a**): 273 K–283 K; (**b**): 288 K–303 K; (**c**): 308 K–318 K; (**d**): 328 K–338 K) interpreted in terms of Equation (3) (solid line), decomposed into the relaxation contributions $R_{1}^{power-law}\left(\omega \right)$ (dashed line) and $R_{1}^{2D}\left(\omega \right)$ (dashed-dotted line).

The data shown in Figure 1a were analyzed in terms of Equation (3). The outcome of the analysis is shown in Figure 2. Although we started the analysis from the data collected at the lowest temperature (273 K), the shape of the frequency dependencies of the relaxation rates observed at the higher temperatures (Figure 2c,d) gave rise to the assumption of a relaxation contribution associated with 2D translation diffusion. In Figure 2c,d, one can clearly see, in the low-frequency range, the described linear dependence of the relaxation rates on the logarithm of the resonance frequency characteristic of 2D diffusion (under the indicated conditions).

The parameters obtained from the analysis are displayed in Table 1. For all temperatures, the larger contribution to the overall relaxation rates is given by the relaxation term $R_{1}^{power-law}\left(\omega \right)$. The power law factor is very low (from 0.138 at 273 K to 0.038 at 338 K). This means that at the highest temperature, this relaxation contribution is only weakly dependent on the resonance frequency. The prefactor *A* is also listed in Table 1 for completeness; however, one should be aware of its phenomenological character.

**Figure 3:**
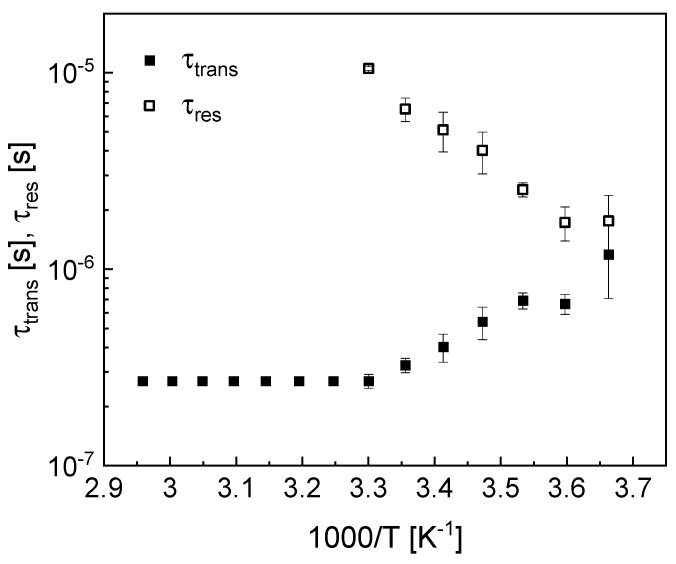
Temperature dependence of the translational correlation time, $\tau_{trans}$, and the residence lifetime, $\tau_{res}$.

Figure 3 shows the temperature dependence of the translational correlation time, $\tau_{trans}$, and the residence lifetime, $\tau_{res}$.

At low temperatures, from 273 K to 183 K (Figure 2a), the relaxation rates at low frequencies deviate from a linear dependence. This is reflected by the shape of the relaxation contribution associated with 2D translation diffusion, $R_{1}^{2D}\left(\omega \right)$. The deviation from the linearity is caused by the relationship between the parameters $\tau_{trans}$ and $\tau_{res}$; the condition $\tau_{res}\gg \tau_{trans}$ is not fulfilled. Following this line, in Figure 2b, this effect continues, becoming progressively less pronounced with increasing temperature as a result of decreasing $\tau_{trans}$ and increasing $\tau_{res}$. Eventually, in Figure 2c,d, the limiting linear behavior is reached. The correlation time $\tau_{trans}$ decreses with increasing temperature (the diffusion process becomes faster, as expected) from 1.19 × 10^−6^ s to 2.70 × 10^−7^ s (by a factor of about 4) and then remains (based on a good approximation) temperature-independent. After figuring out that above 303 K the correlation time $\tau_{trans}$ almost does not change with temperature, the parameter was fixed to the value at 303 K. The residence lifetime, $\tau_{res}$, become longer with increasing temperature, beginning at approximately 2 × 10^−6^ s (at lower temperatures) and reaching values of approximately 1 × 10^−5^ s, after which the quantity cannot be determined (the term ${\left(\frac{\tau_{trans}}{\tau_{res}}\right)}^{2}$ in Equation (2) becomes negligible). The dipolar relaxation constant associated with the 2D diffusion, $C_{trans}$, decreases with increasing temperature by a factor of about 14 (in the temperature range from 273 K to 318 K), indicating that the fraction of BMIM cations involved in the surface diffusion decreases.

## 3. Discussion

As explained in the previous section, the overall ^1^H spin-lattice relaxation rates for [BMIM][Cl]/PC/G2 can be reproduced in terms of two relaxation contributions—one of them representing 2D translation diffusion, the second one following a power law frequency dependence with a very low power law factor. Power law dependencies of spin-lattice relaxation rates on the resonance frequency have been observed for biological macromolecules (proteins) and attributed to the dynamics of the macromolecular backbones affected by cross-linking leading to their partial immobilization. The typical values of the power law factor are in the range of 0.5–0.8 [52,53]. Characteristic power laws have also been predicted and experimentally observed for polymers. The power law factors reach (according to the theory. which was successfully confirmed experimentally) 0.25 for the inter-molecular relaxation contribution for the Rouse dynamics and reptation dynamics, 0.5 for the intra-molecular relaxation contribution for reptation dynamics, 0.625 (5/8) and 0.75 for intra-molecular and inter-molecular relaxation contributions, respectively, in the constrained Rouse dynamics regime [52,54,55,56]. Power laws have also been observed for hydrated, collagen-based artificial tissues, reaching a value of about 0.4 [57]. Following this line, one can expect that in the presence of the gelator (G2), PC forms a partially immobilized network (matrix) whose collective dynamics leads to the $R_{1}^{power-law}\left(\omega \right)$ relaxation contribution. With increasing temperature, the dynamics becomes faster and consequently the frequency dependence of this relaxation contribution becomes less pronounced, which manifests itself with the lower values of α (reaching the limiting behavior of a frequency-independent term). One should note that compared to the power law factors reported in the literature for polymers and proteins, the α values are very low in the present case. The presence of PC enhances the dynamics of BMIM cations in the [BMIM][Cl]/PC mixture, as indicated by the data shown in Figure 1b; the relaxation rates do not show a visible frequency dependence due to the fast dynamics of both components. In the gel matrix, the ions form interconnected domains, while the structure of the matrix poses geometrical restrictions (confinement) yielding the diffusion process highly anisotropic (two-dimensional). The formation of the domains combined with the confinement leads to slowing down of the diffusion process. The correlation time $\tau_{trans}$ can be used to estimate the translation diffusion coefficient, $D_{trans}$, using the relationship $D_{trans}=\frac{d^{2}}{2\tau_{trans}}$. One should, however, keep in mind that BMIM cations are not spherical and the relationship is an approximation. Nevertheless, using 9Å [69] as an effective diameter, one obtains at 273 K a value for $D_{trans}$ of approximately 3.7 × 10^−13^ m^2^/s and approximately 1.5 × 10^−12^ m^2^/s at 303 K and above. It is worth comparing these values with the diffusion coefficient of [BMIM][Cl], being of the order of 2 × 10^−11^ m^2^/s at 298 K [70]. The diffusion process does not become faster with increasing temperature—this indicates the influence of the confinement on the ion mobility. It is also worth noting the values of the dipolar relaxation constant, $C_{trans}$, decreasing with the increasing temperature. This trend indicates a decrease in the fraction of the ionic liquid involved in the 2D translation diffusion or changes in the organization of the ionic domains. Eventually, one should comment on the residence lifetime, $\tau_{res}$, becoming longer with increasing temperature. This finding might be counterintuitive; however, it is supported by the experimental data. The linear dependence of the relaxation rates on the logarithm of the resonance frequency is observed at higher temperatures. One could argue that this is not because of $\tau_{res}$ becoming longer but rather $\tau_{trans}$ becoming shorter. However, the shortening of τ_trans with temperature is only by a factor of about 4, not orders of magnitude. This is also reflected by the shape of the frequency dependencies of the relaxation rates; the change in the “steepness” of the curve can be observed in the range approximately 2 × 10^5^ Hz–1 × 10^6^ Hz within the whole temperature range.

The relaxation studies should not be treated as ultimate proof of the outlined scenario of motion; however, they give insight into dynamics that can hardly be obtained using other methods.

## 4. Materials and Methods

### 4.1. Gel Preparation

**Figure 4:**
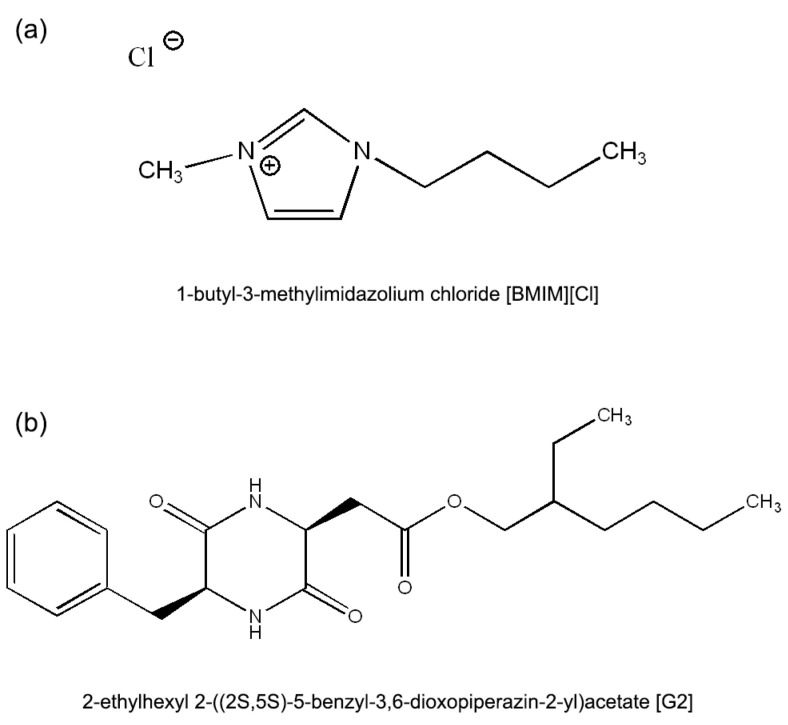
Chemical structures of (**a**) [BMIM][Cl] and (**b**) G2 (low molecular weight gelator).

The propylene carbonate (PC) of spectroscopic grade was purchased from Mreck Company (Warsaw, Poland) and used without further processing. The cyclo(L-beta-2-ethylhexylasparaginyl-L-phenylalanyl) used as the low molecular weight gelator (G2) was synthetized upon order by Synthex Technologies Sp. Z o. o., Toruń, Poland. The chemical composition was verified via NMR and a liquid chromatography–mass spectroscopy (LCMS) analysis (Figures S1 and S2). The 1-butyl-3-methyl-imidazolium chloride ionic liquid ([BMIM][Cl]) was purchased from Merck Company and used without further purification. The chemical structures of the used ionic liquid and low molecular weight gelator are depicted in Figure 4.

The supramolecular ionic gel was prepared according to the following procedures. First, the 1 M solution of [BMIM][Cl] in PC was prepared by dissolving 236.3 mg of [BMIM][Cl] in 1.2 g of PC and continuously stirring the mixture for 15 min at 50 °C. The ionic gel sample was obtained by dissolving 300 mg of gelator G2 in 1 mL of [BMIM][Cl]/PC solution upon stirring for 15 min at 100 °C, followed by cooling at room temperature for 1 h. As a result, we obtained a transparent yellowish gel sample. To verify the gel phase, the samples were subjected to the “inverse tube” and shaker test. Upon testing, the shape and integrity of the gel samples were confirmed. The shaker test was performed with use of a Vortex shaker at 1000 rpm for 60 s, and no change in the sample was observed after the test. The reversibility of the gel to sol phase transition was confirmed by performing subsequent heating and cooling cycles. At temperatures above 80 °C, the sample turned to a liquid state, showing bulk flow behavior; upon cooling at room temperature, it showed solid-like behavior. After each heating–cooling cycle, the “inverse tube” and shaker tests were performed, showing the perfect reproducibility of the sample behavior. The density of the prepared gel was determined to be 1.157 g/cm^3^.

### 4.2. FFC NMR Relaxometry

The ^1^H spin-lattice relaxation experiments were performed using a Spin Master 2000 FFC relaxometer from Stelar Company, Mede, Italy, in the frequency range of 10 kHz to 20 MHz versus temperatures ranging from 273 K to 338 K, stabilized with an accuracy rate of 0.5 K. The relaxation process turned out to be a single exponential over the entire frequency and temperature ranges. The signal from the solid gelator matrix is undetectable because of the fast relaxation characteristics of solids at low and moderate frequencies. Consequently, the detected relaxation process is associated with ^1^H nuclei of [BMIM] [Cl] and PC.

To determine the spin-lattice relaxation rates, ^1^H magnetization decay and recovery curves were recorded as a function of the delay time tau. The delay time tau was incremented from 0.01 s to 4 times the value of the spin-lattice relaxation time in 16 steps. The repetition time between each increment was equal to 5 times the value of the spin-lattice relaxation time, and 4 accumulation stages were applied.

The data analysis was performed using OriginPro 2021. 

## 5. Conclusions

^1^H spin-lattice relaxation experiments were performed for an ionogel composed of [BMIM][Cl] and PC (using G2 as the gelator) in the frequency range of 10 kHz to 20 MHz and the temperature range of 273 K to 338 K. This extensive data set was interpreted in terms of a model assuming two relaxation contributions. A scenario of motion was proposed in which the dynamics of the partially immobilized PC matrix is described by a power law dependence of the relaxation rates on the resonance frequency (with a low power law factor), while the movement of [BMIM] cations is highly anisotropic (associated with a relaxation contribution expressed in terms of spectral density functions characteristic of 2D diffusion). It turned out that the correlation times characterizing the translation diffusion vary approximately between 10^−6^ s and 3 × 10^−7^ s, yielding a corresponding diffusion coefficient of the order of 10^−13^ m^2^/s–10^−12^ m^2^/s. The residence lifetime on the matrix surfaces was estimated as being of the order of 10^−6^ s (at low temperatures) and then increasing with temperature.

## Figures and Tables

**Table 1:** Parameters obtained from the analysis of the ^1^H spin-lattice relaxation data for [BMIM][Cl]/PC/G2 in terms of Equation (3).

**Temp. [K]**	$C_{trans}$ **[Hz^2^]**	$\tau_{trans}$ **[s]**	$\tau_{res}[s]$	α	A
273	(2.93 ± 0.33) × 10^5^	(1.19 ± 0.48) × 10^−6^	(1.76 ± 0.61) × 10^−6^	0.138 ± 0.038	31.7 ± 1.7
278	(2.66 ± 0.18) × 10^5^	(6.67 ± 0.77) × 10^−7^	(1.73 ± 0.34) × 10^−6^	0.132 ± 0.006	25.5 ± 2.3
283	(2.46 ± 0.23) × 10^5^	(6.93 ± 0.65) × 10^−7^	(2.54 ± 0.21) × 10^−6^	0.127 ± 0.003	21.6 ± 1.0
288	(2.21 ± 0.17) × 10^5^	(5.40 ± 1.02) × 10^−7^	(4.02 ± 0.97) × 10^−6^	0.123 ± 0.005	17.3 ± 1.2
293	(2.15 ± 0.19) × 10^5^	(4.02 ± 0.65) × 10^−7^	(5.12 ± 1.17) × 10^−6^	0.122 ± 0.005	14.4 ± 1.1
298	(2.01 ± 0.23) × 10^5^	(3.25 ± 0.27) × 10^−7^	(6.53 ± 0.89) × 10^−6^	0.097 ± 0.003	8.5 ± 0.4
303	(2.02 ± 0.15) × 10^5^	(2.70 ± 0.22) × 10^−7^	(1.05 ± 0.22) × 10^−5^	0.090 ± 0.004	6.5 ± 0.4
308	(1.81 ± 0.04) × 10^5^	2.70 × 10^−7^	-	0.080 ± 0.003	4.7 ± 0.2
313	(1.34 ± 0.03) × 10^5^	2.70 × 10^−7^	-	0.071 ± 0.002	3.4 ± 0.1
318	(1.34 ± 0.08) × 10^5^	2.70 × 10^−7^	-	0.067 ± 0.004	2.8 ± 0.1
323	(8.20 ± 0.25) × 10^4^	2.70 × 10^−7^	-	0.068 ± 0.003	2.6 ± 0.1
328	(5.85 ± 0.22) × 10^4^	2.70 × 10^−7^	-	0.055 ± 0.003	1.9 ± 0.1
333	(3.45 ± 0.24) × 10^4^	2.70 × 10^−7^	-	0.051 ± 0.003	1.7 ± 0.1
338	(2.18 ± 0.19) × 10^4^	2.70 × 10^−7^	-	0.038 ± 0.003	1.2 ± 0.1

## Data Availability

The raw data supporting the conclusions of this article are available at https://doi.org/10.5281/zenodo.15360409.

## Supplementary Materials

The following supporting information can be downloaded at https://www.mdpi.com/article/10.3390/molecules30122598/s1: Figure S1. ^1^H NMR spectra of the gelator cyclo(L-beta-2-ethylhexylasparaginyl-L-phenylalanyl) in DMSO. Figure S2. LCMS spectra of the gelator cyclo(L-beta-2-ethylhexylasparaginyl-L-phenylalanyl.
