# Supplementary material for: Dynamics of Supramolecular Ionic Gels by Means of Nuclear Magnetic Resonance Relaxometry—The Case of [BMIM][Cl]/Propylene Carbonate Gel
Source: Molecules. 2025 Jun 15;30(12):2598. doi: 10.3390/molecules30122598 (PMC12195630; doi:10.3390/molecules30122598)
Supplement: Supplementary file 1 [file molecules-30-02598-s001.zip › molecules-3647941-supplementary.pdf]

# Supplementary Material

# Dynamics of Supramolecular Ionic Gels by Means of Nuclear Magnetic Resonance Relaxometry—The Case of [BMIM][Cl]/Propylene Carbonate Gel

**Michał Bielejewski [1], Robert Kruk [2] and Danuta Kruk [3,*]**

1 Institute of Molecular Physics, Polish Academy of Sciences, M. Smoluchowskiego 17, 60-179 Poznań, Poland; michal.bielejewski@ifmpan.poznan.pl
2 Department of Chemistry, University of Warmia and Mazury in Olsztyn, Plac Łódzki 4, 10-957 Olsztyn, Poland; robert.kruk@uwm.edu.pl
3 Department of Physics and Biophysics, University of Warmia and Mazury in Olsztyn, Michala Oczapowskiego 4, 10-719 Olsztyn, Poland
* Correspondence: danuta.kruk@matman.uwm.edu.pl

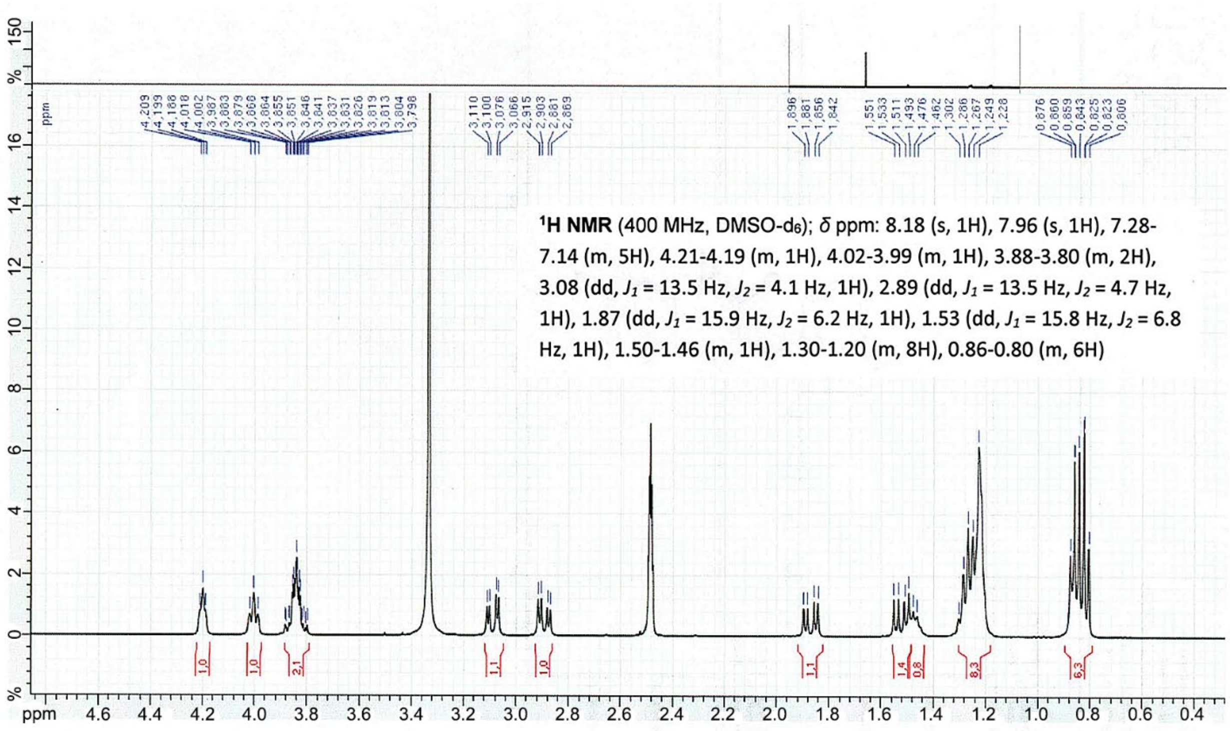


**Figure S1.** $^1$H NMR spectra of the gelator cyclo(L-beta-2-ethylhexylasparaginyl-L-phenylalanyl) in DMSO.

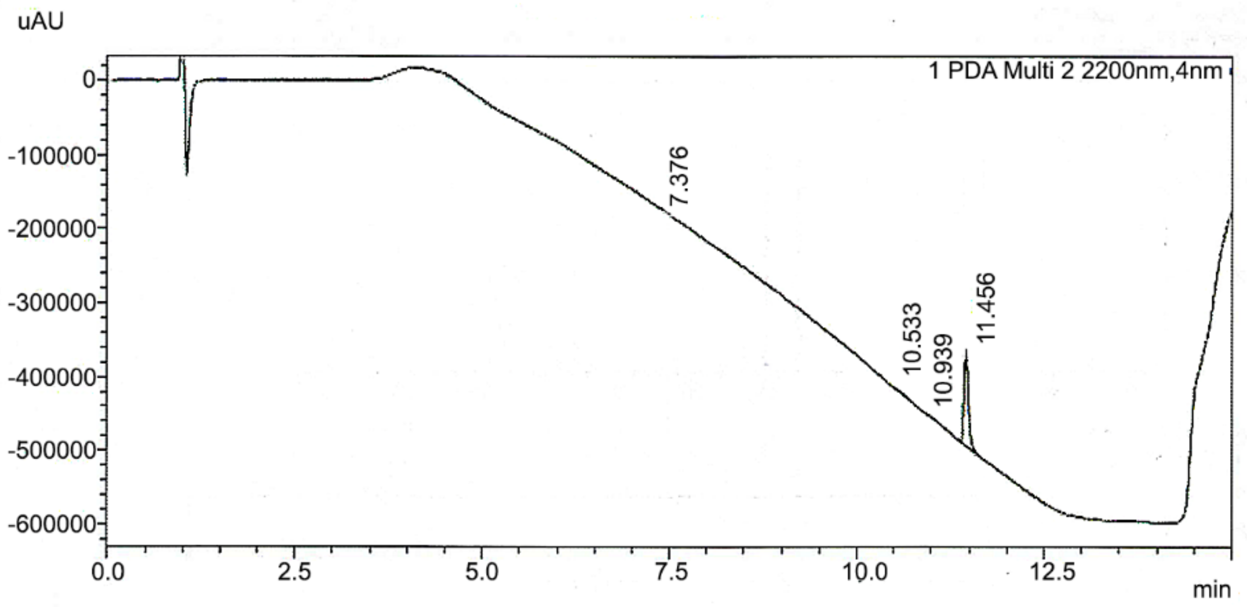


Peak Table

PDA Ch2 220nm

| Peak# | Ret. Time | Area | Area% |
|---|---|---|---|
| 1 | 7.376 | 3153 | 0.593 |
| 2 | 10.533 | 996 | 0.187 |
| 3 | 10.939 | 2598 | 0.489 |
| 4 | 11.456 | 525006 | 98.731 |
| Total | | 531754 | 100.000 |

**Figure S2.** LCMS spectra of the gelator cyclo(L-beta-2-ethylhexylasparaginyl-L-phenylalanyl).
